# A comprehensive metatranscriptome analysis pipeline and its validation using human small intestine microbiota datasets

**DOI:** 10.1186/1471-2164-14-530

**Published:** 2013-08-02

**Authors:** Milkha M Leimena, Javier Ramiro-Garcia, Mark Davids, Bartholomeus van den Bogert, Hauke Smidt, Eddy J Smid, Jos Boekhorst, Erwin G Zoetendal, Peter J Schaap, Michiel Kleerebezem

**Affiliations:** 1 TI Food and Nutrition (TIFN), P.O. Box 557, 6700 AN, Wageningen, The Netherlands; 2 Laboratory of Microbiology, Dreijenplein 10, Wageningen 6703 HB, The Netherlands; 3 Laboratory of System and Synthetic Biology, Wageningen University, Dreijenplein 10, 6703 HB, Wageningen, The Netherlands; 4 Laboratory of Food Microbiology, Wageningen University, P.O. Box 8129, 6700 EV, Wageningen, The Netherlands; 5 Host-Microbe Interactomics Group, Wageningen University, P.O. box 338, 6700 AH, Wageningen, The Netherlands; 6 Centre for Molecular and Biomolecular Informatics, Radboud University Medical Centre, Nijmegen, The Netherlands; 7 NIZO Food Research B.V, P.O. Box 20, 6710 BA, Ede, The Netherlands

**Keywords:** Metatranscriptome, Bioinformatic pipeline, Human small intestine microbiota, Illumina sequencing, Single-end reads, Paired-end reads, COG, KEGG, Metabolic pathways

## Abstract

**Background:**

Next generation sequencing (NGS) technologies can be applied in complex microbial ecosystems for metatranscriptome analysis by employing direct cDNA sequencing, which is known as RNA sequencing (RNA-seq). RNA-seq generates large datasets of great complexity, the comprehensive interpretation of which requires a reliable bioinformatic pipeline. In this study, we focus on the development of such a metatranscriptome pipeline, which we validate using Illumina RNA-seq datasets derived from the small intestine microbiota of two individuals with an ileostomy.

**Results:**

The metatranscriptome pipeline developed here enabled effective removal of rRNA derived sequences, followed by confident assignment of the predicted function and taxonomic origin of the mRNA reads. Phylogenetic analysis of the small intestine metatranscriptome datasets revealed a strong similarity with the community composition profiles obtained from 16S rDNA and rRNA pyrosequencing, indicating considerable congruency between community composition (rDNA), and the taxonomic distribution of overall (rRNA) and specific (mRNA) activity among its microbial members. Reproducibility of the metatranscriptome sequencing approach was established by independent duplicate experiments. In addition, comparison of metatranscriptome analysis employing single- or paired-end sequencing methods indicated that the latter approach does not provide improved functional or phylogenetic insights. Metatranscriptome functional-mapping allowed the analysis of global, and genus specific activity of the microbiota, and illustrated the potential of these approaches to unravel syntrophic interactions in microbial ecosystems.

**Conclusions:**

A reliable pipeline for metatransciptome data analysis was developed and evaluated using RNA-seq datasets obtained for the human small intestine microbiota. The set-up of the pipeline is very generic and can be applied for (bacterial) metatranscriptome analysis in any chosen niche.

## Background

The microbial ecology of the human small intestine is of interest since it is the first site where intestinal microbes interact with ingested food components [1]. Due to poor physical accessibility, there is only limited knowledge of the microbial gene functions and metabolic pathways that are operating in the small intestine microbiota. Powerful high throughput sequence-driven metagenomic approaches have emerged to study the genetic potential of complex gut microbial communities [2,3]. Metagenome analyses of microbial samples derived from the human small intestine have previously identified genes and corresponding pathways involved in transport and metabolism of simple carbohydrate substrates to be enriched in the small intestine microbiota, suggesting their importance for the functioning of this microbial ecosystem [4]. However, elucidation of the actual activity and metabolic role of individual microbial members in the small intestine is still limited.

Transcript abundance as a proxy for activity may complement the DNA-based analyses by identifying the metabolic activity patterns of individual microbial members and by unraveling the community responses to changing environmental conditions [5-7]. Metatranscriptome analysis at a small scale using a cDNA cloning and sequencing approach already demonstrated that the genes and functional categories that are enriched in the small intestine metagenome, were also among the highest expressed functions [4]. The application of mRNA enrichment procedures in combination with next generation sequencing (NGS) technologies on pure cultures has clearly established that in depth analysis of transcriptomic landscapes are now feasible [8-11] and was applied to microbial communities of marine [12,13], soil [14,15], and human gastrointestinal tract [16,17] origins. The two most frequently used high throughput RNA sequencing (RNA-seq) technologies for metatranscriptome analysis are provided by Roche-454 FLX Titanium [12,16] and Illumina [17], which differ in the length and the number of sequence reads generated. Current Illumina protocols generate single-end sequencing reads with a shorter read length, but with a greater depth of analysis as compared to Roche-454 technology [18]. Notably, to extend the Illumina read-length, paired-end sequencing technology can be applied to increase the information content and to enhance the identification of expressed gene functions in complex communities [19].

Both RNA-seq technologies mentioned above provide large datasets of great complexity, which require a reliable bioinformatics pipeline to effectively convert the initial data into biologically relevant information. Goncalves *et al.*[20] constructed an *R*-based pipeline for pre-processing, quality assessment, and expression estimation of RNA sequencing datasets. Analogously, a bioinformatics analysis pipeline for Roche 454 sequencing technology derived metatranscriptome of soil and seawater samples was described by Gilbert *et al*. [5], which includes removal of low quality reads and reads originating from rRNA sequences, followed by functional annotation based on the predicted open reading frames (ORFs) and comparison between datasets. However, several steps were not described in detail and although this methodology appears to be appropriate for processing and interpreting of 454 datasets, it may not be suitable for the massive amounts of short-read length reads that are present in Illumina generated datasets.

Here we present a reliable pipeline that can process Illumina-RNA-seq metatranscriptome data by linking the sequence reads to reference gene databases, their assigned functions, and predicted phylogenetic origin. The information retrieved from the processed data can be employed to obtain comprehensive biological insights in the ecosystem’s activity patterns. We employed this pipeline for the primary interpretation of the activity patterns of the human small intestine microbiome using effluent samples obtained from individuals with an ileostoma.

## Results and discussion

Two RNA samples designated A and B were extracted from effluent samples of two healthy ileostomy subjects with an overall yield of 435 and 30 μg of total RNA per ml of effluent, respectively. The difference in yield correlated with a more than 10-fold higher total bacterial community density per ml in sample A relative to sample B, which was determined by qPCR targeting the 16S rRNA gene (data not shown). The RNA samples had reasonable RNA-quality scores (16/23S rRNA ratio) of 1.5 and 0.7 for samples A and B, respectively (Additional file 1: Figure S1). Although sample B was estimated to contain lower quality RNA compared to sample A, the RNA size-profile of sample B still lacked the intense degradation RNA peaks that are typical for poor-quality RNA (Additional file 1: Figure S1), and thereby meet the criteria set for RNA quality [21]. From the total RNA, mRNA was enriched using a bead-based selective capture procedure, which was previously shown to have a limited impact on the mRNA composition [11]. However, as this procedure is based on 16S and 23S rRNA specific capture probes, rRNA removal efficiency varies between bacterial species. The mRNA enrichment procedure resulted in approximately 70% removal of the total ribosomal RNA based on the quantity of enriched mRNA that was recovered (Nanodrop measurement).

**Figure 1:**
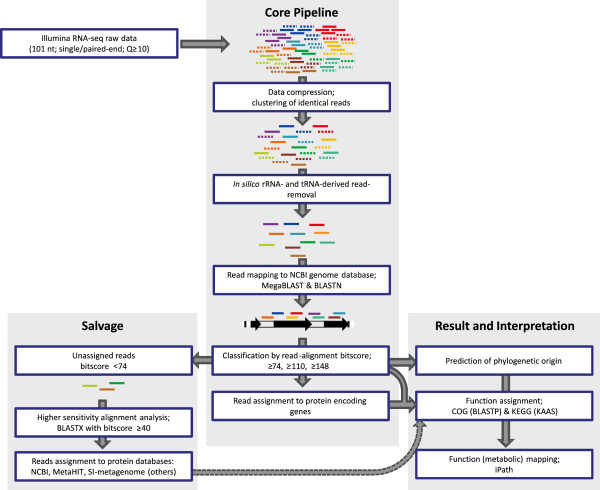
**Flow diagram of the bioinformatics analysis pipeline.** The rRNA/tRNA reads were removed from the unique Illumina reads using SortMeRNA software followed by BLASTN alignment to NCBI and SILVA ribosomal databases. The mRNA reads are assigned to the prokaryote genomes of NCBI using MegaBLAST followed by BLASTN, followed by classification according to alignment bit scores using a minimum bit score of 148 and 110 for prediction of phylogenetic origin at genus and family level, respectively. The genome assigned reads were classified into protein encoding or non-coding reads, followed by COG and KEGG functional annotation and metabolic mapping. Additional functional assignment was performed for evaluation purposes by assigning 10% of randomly selected unassigned reads (bit score ≤74) to the NCBI protein database followed by MetaHIT and SI metagenome databases using BLASTX (see methods for details).

Both single- and paired-end Illumina cDNA libraries had an insert size ranging between 200-300 bp. Two independent single-end cDNA libraries of sample A were constructed and sequenced, yielding datasets ‘A’ that contained ~29.7 million reads and ‘A-rep’ that was sequenced at 3-fold lower depth and contained ~9 million reads. The mRNA-enriched RNA of sample B was used to construct a paired-end sequencing library, of which the sequencing generated approximately 42.2 million read-pairs. Both single and paired-end sequencing reads had a read-length of 101nt. The paired-end sequencing dataset of sample B was split in two individual datasets arbitrarily designated B-left and B-right, corresponding to the forward and reverse Illumina reads, respectively. The resulting four datasets (A, A-rep, B-left, B-right) were used for the development and validation of a bioinformatics analysis pipeline (Figure 1), and for primary functional analyses of the resulting activity patterns of the human small intestine microbiota.

### Development of the RNA-seq bioinformatics analysis pipeline

The overall quality score of the raw sequence reads was very high. The vast majority of the reads had average Phred scores of more than 30 (> 63% for datasets A and > 78% for datasets B; Additional file 2: Figure S2). To avoid unreliable results, most metatranscriptome studies to date only analyzed sequence reads with high average Phred scores [5,16]. However, a recent analysis of RNA-seq datasets obtained for *Bacillus subtilis* str. 168 [22] demonstrated that lower-quality RNA-seq reads can still be reliably mapped to specific regions of the strain’s genome, indicating that removal of lower-quality reads may lead to loss of biologically relevant information. Since the aim of our study is to assign function and phylogeny information to individual reads, rather than higher-stringency multi-read procedures like re-sequencing or assembly, we employed a minimum average Phred score of 10 as a cut-off filter in primary data-processing steps (Figure 1). Using this threshold, less than 5% of the total reads was removed from the RNA-seq datasets used in this study.

The massive Illumina RNA-seq datasets may contain repeated sequence-reads that align to identical genetic loci. These reads are likely deriving from highly expressed and/or highly conserved sequence regions of strains and species in the community, such as the rRNA genes [23,24]. To avoid inefficient and repetitive mapping of these repeated reads, and to reduce the dataset’s complexity, identical reads within a dataset were clustered (Additional file 3: Table S1). This compression strategy significantly reduced the complexity of the dataset, yielding approximately 12–13 million unique-reads for the datasets A, B-left, and B-right and 4 million unique-reads for the lower-depth dataset A-rep (Additional file 3: Table S1), which were subjected to further analysis. The clustering of identical reads was executed for compression purposes only, and transcript-abundance calculations employed the actual read-abundance of the clustered identical reads.

#### Removal of rRNA and tRNA sequences

Although an mRNA enrichment step was performed prior to sequencing-library construction, rRNA removal efficiencies are generally incomplete. Therefore, removal of reads originating from rRNA and tRNA sequences is a compulsory procedure when dealing with RNA-seq datasets from bacteria [5]. In addition, the Illumina data may still contain a fraction of reads originating from phiX spike-in control sequences and Illumina adaptor sequences that should also be removed. To this end, iterative filtering steps were employed to remove 80-86% of the reads on basis of alignment to rRNA/tRNA (bacterial, archaeal and eukaryotic origin), to phiX, and to the Illumina adaptor sequences (Additional file 3: Table S2). To test the performance of this filtering step, the paired-end reads of dataset B were filtered separately, revealing a virtually identical (98.6% identity) removal efficiency from the read pairs of the B-left and B-right datasets.

Because the oligonucleotides employed in the mRNA enrichment procedure applied here are targeting the 5’- and 3’-terminal ends of the 16S and 23S rRNA molecules, the efficacy of the procedure largely depends on the fraction of intact total rRNA in the sample. Therefore, the relatively high amount of rRNA derived reads that remained in the RNA-seq datasets suggested the presence of (partially) degraded rRNA fragments in the original total RNA samples that were not captured by the rRNA probes [25]. The reads coding for rRNA and tRNA sequences were analyzed for their overall taxonomic composition. The results indicated that more than 99% of these reads originated from prokaryotes and less than 1% could be assigned to a eukaryotic origin (mostly yeasts belonging to the *Candida* and *Saccharomyces* genera). Based on the low number of eukarya derived sequences in the RNA-seq datasets, further analysis was focussed on assigning the reads of prokaryotic (mainly bacterial) origin.

#### Read-assignment to the reference genome database

The RNA-seq reads that passed the rRNA/tRNA filter are assumed to represent mRNA transcripts of bacterial origin (mRNA reads) that are largely expected to derive from protein encoding genes that are transcribed, but could also derive from intergenic non-coding genomic regions. Phylogenetic and functional assignments were obtained by directly aligning the mRNA reads to the complete NCBI prokaryote genome database that included draft genomes.

**Figure 2:**
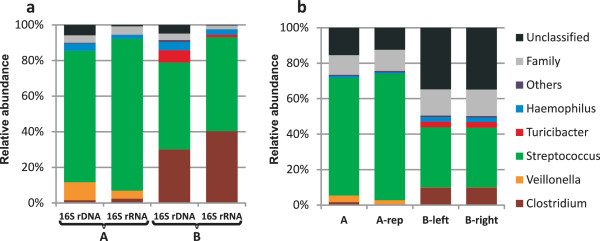
**Phylogenetic profiling of datasets A and B.** Phylogenetic profiling of detected bacterial taxa for 16S rDNA and rRNA sequences obtained from pyrosequencing **(a)** and for mRNA reads obtained from Illumina sequencing **(b)**. Both 16S and mRNA reads were classified into genus (colour key), or family (light grey), classified reads and the remaining unclassified reads (dark grey), based on the applied cut off (see methods). Only genera that contribute at least 2% to one of the profiles were represented. Separate phylogenetic profiling at genus level using 16S and mRNA reads of both datasets is presented in figure S4.

According to simulation experiments (see Additional file 4 and Additional file 5: Figure S3A), minimum bit score thresholds of 148 and 110 can be used for phylogenetic assignments at genus- and family-level, both with > 80% confidence level. Respectively 73% and 50% of the mRNA reads of datasets A and B aligned with sequences in the NCBI prokaryote genome database with a bit score of 148 or higher. The phylogenetic distribution of these genus-assigned mRNA reads revealed that 70% and 34% of the mRNA reads were assigned to the genus *Streptococcus* in the RNA-seq datasets A and B, respectively. Both datasets also contained mRNA reads that were assigned to the genus *Veillonella*, which appeared to be more abundant in the dataset A (3%) as compared to dataset B (0.2%). Inversely, mRNA reads that were assigned to the *Clostridium* and *Haemophilus* genera were more abundant in dataset B (9% and 2.5%, respectively) compared to dataset A (2% and 1%, respectively) (Figure 2). Notably, *Turicibacter*-assigned mRNA reads were only encountered in the dataset B (3%). These observations illustrate the subject specificity of the small intestinal microbiota ecosystem activity profile. A similar conclusion was also reached in previous studies that described the human small intestine microbiota composition, revealing relatively consistent high abundances of *Streptococcus* spp. in different individuals and a more variable relative abundance of species belonging to the genera of *Veillonella* and *Clostridium*[4,26,27]. Highly similar phylogenetic distributions at genus-level were obtained for datasets A and A-rep, while the separate analysis of the B-left and B-right datasets generated a virtually identical phylogenetic profile (Figure 2; Additional file 6: Figure S4).

The mRNA reads with an alignment bit-score between 110 and 148 could be confidently assigned at family level and represented 11% and 15% of the reads in datasets A and B, respectively. The family-level assignments identified in dataset A were dominated by the family of the Streptococcaceae that captured approximately 85% of the family-assigned reads. By contrast, 65% of the family-assigned reads identified in dataset B belonged to the Clostridiaceae family, supporting the composition difference in these samples that was also apparent from the genus-assigned mRNA reads. The mRNA reads in datasets A and B that had alignment bit-scores below 110 (between 12 and 35% of the total mRNA reads) were grouped as unclassified reads (Figure 2).

The microbiota composition of the samples A and B was also determined using a 16S rRNA gene and transcript targeting approach, using 16S rDNA and 16S rRNA pyrosequencing. Of the total sequence reads obtained in this analysis, between 90 and 97% could be assigned to genus level using the RDP classifier with a 80% confidence threshold [28]. A remarkable overall similarity of the relative genus abundances was observed for the phylogenetic reconstruction of the small intestinal microbiota ecosystem on basis of the 16S (rDNA and rRNA) datasets and the phylogenetic assignments of the mRNA reads (Additional file 6: Figure S4). This observation is in clear contrast with the low correlation observed between the total and active fractions of the fecal microbiota community [29,30], indicating that compared to the fecal microbiota, the human small intestine ecosystem displays more congruency in microbial composition and activity (overall and specific) patterns.

Notably, the mRNA derived phylogenetic ecosystem reconstruction contained a substantial amount (>24%) of reads that could not be assigned at genus level, while the 16S-based sequences could nearly all be assigned at genus level (>90%) (Figure 2). This illustrates the superiority of the 16S rRNA gene as a phylogenetic marker for microbiota composition profiling, which is probably due to the lower coverage of the gene-sequence space within the prokaryotic genome database as compared to the 16S rRNA sequence database.

**Figure 3:**
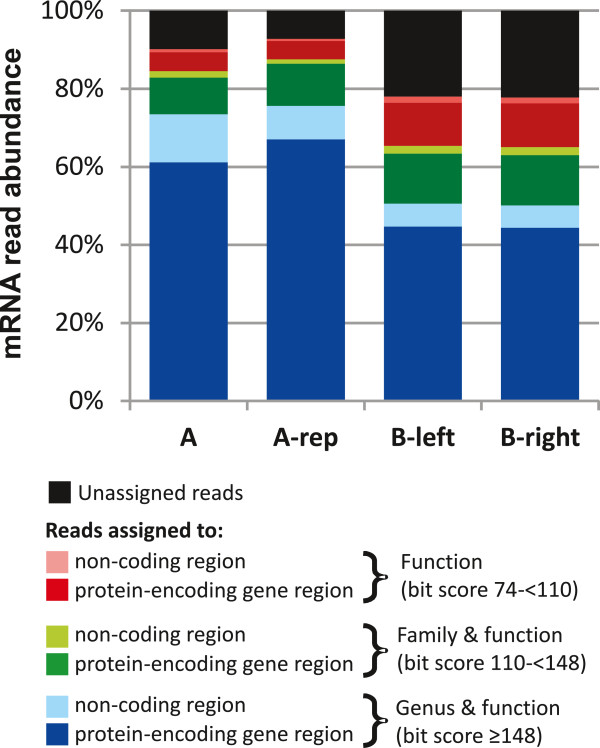
**Distribution of mRNA reads assignment.** The mRNA reads were assigned to the reference genome database and classified based on their alignment to protein-encoding genes (dark bars) or non-coding (light bars) regions. Based on alignment bit score of mRNA reads to the genome, the reads can obtain phylogenetic and functional identification at genus (blue) and family (green) levels with a minimum bit score of 148 and between 110 and 148, respectively; while the reads with an alignment bit score between 74 and 110 only obtained functional assignments (red). The unassigned reads were represented in black. The specific read numbers that belong to each classification are presented in table S4.

All read alignments with minimum bit score of 74 or higher could reliably (>95% confidence) be assigned to a COG-based function (see Additional file 4 and Additional file 5: Figure S3A). Using this minimum bit score threshold, 78 to 93% of the mRNA reads (Figure 3, Additional file 3: Table S4) could be assigned to homologous loci in bacterial genomes using MegaBLAST (65-85%) and BLASTN (8-14%). This relatively high hit-frequency illustrates that the NCBI genome database provides a good representation of the functional diversity encountered in the human small intestine ecosystem. However, it should be noted that samples from other ecosystems may be less well represented in this database.

#### Classification of aligned mRNA reads to protein and non-protein encoding gene regions

Bacterial genomes commonly have a relatively high coding density, but also contain non-coding or intergenic regions [31,32]. Therefore, not all mRNA reads in the dataset are necessarily derived from transcripts of protein-coding genes, but may also represent non-coding 5’-, or 3’-untranslated regions of gene transcripts or specific non protein-coding RNA molecules. To restrict the analysis to mRNA reads that contain functional gene-expression information, the reads assigned to protein-encoding genes were selected among the genome-assigned reads (see methods). This procedure captured more than 84% of the total genome assigned reads, illustrating that a relatively limited percentage of mRNA reads aligned with intergenic or non-coding regions in the genome database (Figure 3, Additional file 3: Table S4) [33]. Although mRNA reads were aligning with 41 to 99 thousand different genes per dataset, only a fraction of these assignments were considered to be significant. Significant gene expression identification was determined by ranking the genes based on the number of reads that were assigned to the gene, followed by selecting the top ranking genes that captured 95% of the assigned mRNA reads. Application of this threshold limits the number of gene-transcript identifications to approximately 10 to 22 thousand genes per dataset (Table 1), supporting that this restrictive step removed numerous spuriously identified gene-transcripts.

**Table 1:** The number of significant gene assigned reads and significantly identified genes

	**A**	**A-rep**	**B-left**	**B-right**
Number of identified genes	65,594	41,269	99,145	97,875
Number of significantly identified genes	13,878	10,407	22,237	22,223
Number of gene assigned reads	3,238,544	1,400,017	4,067,222	4,048,999
Number of significant gene assigned reads	3,074,411	1,329,217	3,856,962	3,841,245

For genes that were predicted to originate from a specific taxonomic origin, full-length mRNA read coverage strongly supports the expression of the encoded function. Therefore, the coverage of each gene detected by mRNA read mapping was determined, revealing that approximately 40% of all genes identified within the datasets A, B-left and B-right have more than 80% of their sequence length covered (orange boxes in Additional file 7: Figure S5). The high coverage gene-sets are expected to represent the highly expressed functions deriving from the dominant microbial genera in the ecosystem, and encompassed translation associated functions, glycolytic enzymes, and components of carbohydrate phosphotransferase systems (PTS). Due to a three-fold lower sequencing depth, gene coverage is lower for dataset A-rep (Additional file 7: Figure S5). Nevertheless, only 25% less genes were identified in dataset A-rep compared to dataset A (Table 1), implying that the lower depth of metatranscriptome analysis performed for dataset A-rep (relative to dataset A) still generated a substantial amount of information with respect to the main microbiome activity patterns (see metabolic pathway mapping below). This observation may specifically be valid for microbial ecosystems of limited phylogenetic complexity, such as the human small intestine, which harbors ‘only’ approximately 5–10 dominant genera, but may not hold true for ecosystems that contain much more complex microbial communities with hundreds of different genera, such as the large intestine microbiota.

### Analysis of unassigned mRNA reads

To further increase the function assignment of the mRNA reads, the more sensitive protein-sequence alignment was qualitatively evaluated using BLASTX [19,34]. To this end, a randomly selected fraction of 10% of the reads that were not assigned to the prokaryotic genome database by nucleotide alignment (Figure 3, Additional file 3: Table S4) were subjected to BLASTX-based alignment to the NCBI protein database. This analysis revealed that approximately 50 and 70% of the unassigned reads could be significantly assigned to proteins in the datasets A and B, respectively. Notably, the majority (between 60-80% of the protein assigned reads) of the proteins that were detected by these alignments had a functional annotation (Additional file 3: Table S5), indicating that this salvage strategy may expand the detected protein-function repertoire. For both datasets the translated mRNA reads were predominantly assigned to proteins derived from the *Clostridium* related species (A: 40% and B: 80%). *Clostridium* genus displays an extensive genetic diversity and the results suggest that the small intestinal members of this genus are less well represented in the prokaryote genome database. Subsequently, the reads that remained unassigned after BLASTX-alignment to the NCBI protein database were BLASTX-aligned to the 3.3 million genes of the human fecal metagenome database [35] and the approximately 170,000 genes of the human small intestinal metagenome database [4]. These alignments allowed the assignment of a further 10% and 16% of reads derived from datasets A and B, respectively. Overall, the finally remaining unassigned fraction of mRNA reads was approximately 3% of the total putative mRNA-derived reads in the datasets. These reads could represent novel bacterial genes [36] that are not represented in the databases used, or could represent putative mRNA sequences originating from eukaryotes, that may be related to the eukaryal rRNA sequences that were identified at a low frequency in these datasets during the rRNA filtering step (see removal of rRNA and tRNA sequences above), or finally, they may also be the result of sequencing artifacts.

### Reproducibility and paired-end versus single-end sequence analyses

To establish reproducibility of the RNA-seq approach, a technical duplicate sequencing dataset was generated for the sample obtained from subject A (albeit at lower sequencing depth). Comparison of the number of reads that were significantly assigned to genes for the two sequence datasets of sample A revealed a highly significant Pearson correlation of 0.973 (p<0.01), supporting the high reproducibility of the RNA-seq approach as has also been reported in previous studies [37-39]. Consistency in read assignment was also apparent for the paired-end reads, where 91% of the paired mRNA reads independently aligned with sequences from the same genus (Additional file 8: Figure S6). However, only 79% of the paired-end reads aligned to the same species, which is in line with results obtained from the simulated reads (Additional file 5: Figure S3). Due to high levels of sequence similarity within species of the same genus, the simulated read analysis (Additional file 5: Figure S3A) predicted that a reliable phylogenetic assignment could only be achieved at genus level. In terms of function assignment (see COG functional analysis below), a near-perfect congruency was observed between the mate-pairs (Pearson correlation coefficient: 1.00; p<0.01), implying that no additional functional information can be obtained from paired-end, relative to single-end sequencing methods.

### Functional analysis and metabolic pathways mapping

To obtain insight in the functional properties of the human small intestinal microbiota, significantly detected genes were assigned to COGs [40] for functional analysis and linked to KEGG orthologs [41] for pathway identification. For approximately 89% of the genes that were detected, their encoded proteins could be assigned to a specific COG, of which between 80-90% have a functional annotation (Additional file 3: Table S5), whereas approximately 69% could be assigned to a KEGG-Orthology (KO) identifier. The overall fraction of the total mRNA reads that were captured within those COG and/or KEGG annotated genes were 86 to 94% and 71 to 81% for COG and KEGG assignments, respectively.

**Figure 4:**
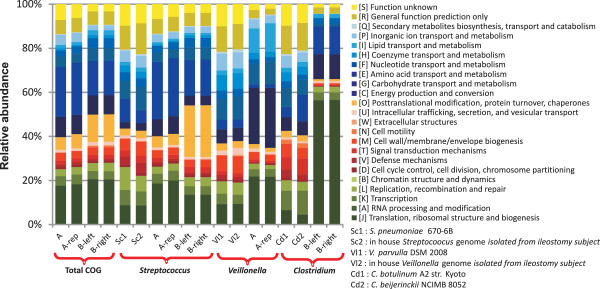
**Distribution of COG functional categories for datasets A and B.** Total COG distribution profiles were analyzed using reads with a minimum alignment bit score of 74. Genus specific COG distributions of the two most dominant genera were obtained using a minimum alignment bit score of 148. The COG distribution of the genes annotated in the complete genomes of representative (intestinal and non-intestinal) genomes of strains belonging to the three genera displayed here were included for comparison purposes.

The majority of the COG function-assigned transcripts that were detected belong to functional categories “information storage and processing” (predominantly sub-category “translation, ribosomal structure and biogenesis”) and “metabolism”. (predominantly subcategory “carbohydrate transport and metabolism”) (Figure 4). These findings suggest that the microbial communities are growing and metabolically active. Notably, in a metatranscriptome study that targeted the fecal microbiota [16], a predominance of the same functional categories was observed.

As anticipated, similar COG identifiers (IDs) were assigned to the protein-homologues of the technical replicate datasets (A and A-rep), which is also clearly apparent from the similar COG category distribution of both datasets derived from sample A (Figure 4). Analogously, in the datasets obtained for sample B, only 1.5% of the read pairs were assigned to different COG IDs (Additional file 8: Figure S6). Remarkably, comparison of the COG IDs detected in datasets A and B revealed a significant similarity between the two samples (Pearson correlation of 0.713; p<0.01). However, no similarity was observed in the comparison of the A and B datasets with respect to the expression levels of each significantly expressed gene (Pearson correlation of −0.001; p>0.05). These observations imply that although these datasets are derived from different microbiomes in terms of species composition and gene expression profiles, the qualitative functional activity profiles are comparable, supporting substantial functional redundancy in the community members.

**Figure 5:**
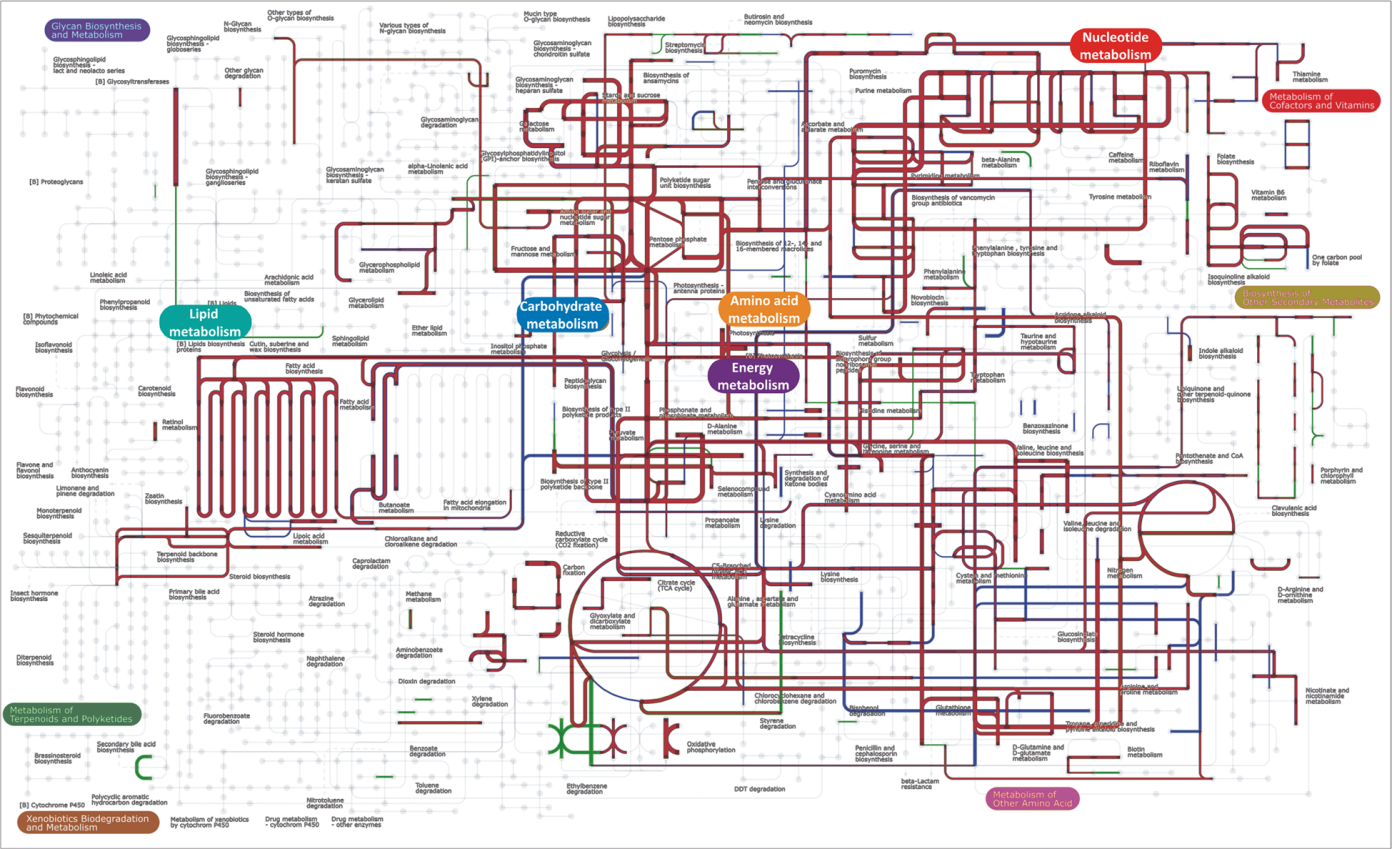
**Metabolic pathways mapping of dataset A and B.** Metabolic pathways belonging to lipid, carbohydrate, energy, nucleotide, and amino acid metabolism were dominantly expressed in both datasets. The majority of the metabolic pathways overlapped between datasets A and B (red lines), while unique pathways for dataset A or B were indicated as green and blue lines, respectively. The line width indicates gene expression levels. Metabolic pathways were generated using iPath v2 based on KEGG annotation of the detected genes.

Annotations using the KEGG database [41] were performed to enable effective metabolic pathway identification using the compatible iPath pathway mapping system [42], which is less well compatible with COG function assignments. Metabolic pathway mapping of the transcript profiles obtained from datasets A and A-rep gave an overall similar result (Additional file 9: Figure S7). Nevertheless, the higher resolution of dataset A allowed for the identification of pathways involved in secondary metabolite production and lipopolysaccharide biosynthesis (Additional file 9: Figure S7). As expected, identical pathway mapping results were obtained for datasets B-left and B-right of sample-B (data not shown). The metabolic mapping of the metatranscriptomic profile of datasets A and B also displayed a high degree of similarity (Figure 5). Both profiles revealed major similarity of pathways related to nucleotide, carbohydrate, amino acid, energy, and lipid metabolism, as well as cofactor and vitamin synthesis. Nevertheless, detailed analysis still allowed detection of differences of pathways related to oxidative phosphorylation and propanoate metabolism which were detected at a much higher level in dataset A, while pathways related to metabolism of specific amino acids were more abundant in dataset B (Figure 5). These differences may reflect ecosystem adaptations to environmental differences such as variation in the dietary composition of subject A and B.

For each dataset, the COG distributions of the mRNA reads that could be assigned to the two most abundant genera for each sample were analyzed to evaluate genus specific functional activity profiles within the ecosystem. These analyses were performed for the genera *Streptococcus*, *Veillonella* and *Clostridium*, using two representative reference genomes of each genus from intestinal and non-intestinal isolation origins (see methods). In comparison with the COG functional category profile of the reference genomes, the COGs assigned to the genera *Streptococcus* (datasets A and B), *Veillonella* (dataset A) and *Clostridium* (dataset B) were enriched for the functional categories “carbohydrate transport and metabolism”, “energy production and conversion”, and “translation, ribosomal structure and biogenesis”, respectively (Figure 4). In addition, the *Streptococcus* assigned COG distribution profile resembled the total COG distribution profile, which is a reflection of the abundance of *Streptococcus*-assigned reads in these datasets (Figure 4). Variation in genus-specific COG distributions observed in both datasets is indicative for the distinct activities that can be observed for the different genera. This finding can be used as a starting point to decipher genus specific metabolic activity as well as the deduction of syntrophic relationships between different genera in the small intestine ecosystem.

## Conclusions

Insight into the functional characteristics of the microbiota residing in different ecosystems can be obtained from metatranscriptome analysis. In this study, a pipeline was developed for the analysis of massive amounts of RNA-seq data, which was evaluated using metatranscriptome datasets obtained from the microbial ecosystem found in the human small intestine. The pipeline enabled the effective removal of rRNA derived sequences that dominated the primary sequence data, despite the implemented mRNA enrichment procedure. This implies that the extend of the functional analysis still can be significantly improved by increasing the efficiency of rRNA removal prior to cDNA library construction. Nevertheless, a substantial number of sequence reads derived from protein-encoding mRNA transcripts that could be assigned to a function and/or to a phylogenetic origin at genus or family level were obtained in this study. Moreover, the phylogenetic origin of specific transcripts and corresponding expressed functions was further substantiated by a global read-coverage of specific genes, such as genes that are highly expressed by dominant microbial members of the ecosystem.

Although in this study, the pipeline was evaluated only for samples derived from the human small intestine, its generic set-up by using a ‘complete’ rRNA database for filtering and the entire NCBI prokaryote genome database for mRNA read-mapping makes it suitable for metatranscriptome analysis of datasets derived from other niches. Nevertheless, the reliability of the assignment of the phylogenetic origin of expressed sequences is dependent on high sequence similarity levels in the alignment with a reference genome database.

This study establishes the reproducibility of the metatranscriptome sequencing approach to unravel the main activity profiles in the ecosystem, despite differences in sequencing depth. It also shows that functional insights and accuracy of the phylogenetic assignment are not significantly improved by the application of paired-end sequencing procedures.

For the small intestine ecosystem, a strong correlation was observed between the community composition (16S rDNA), the overall activity of the community members (16S rRNA), and the specific activity of community members (mRNA). Preliminary functional profiling of the metatranscriptomic landscapes revealed the overall (metabolic) activity profiles belonging to the microbial ecosystem of the human small intestine. This implies that the method presented provides a means to monitor the impact of for example, dietary interventions on the microbiota activity. Moreover, metatranscriptome analysis enabled the discrimination of functional COG expression profiles of specific genera within the ecosystem, which provides a framework for further study aiming to unravel syntrophic relationships that are operating in complex microbial ecosystems.

## Methods

### Ethics statement

The study was approved by the University Hospital Maastricht Ethical Committee, and was conducted in full accordance with the principles of the ‘Declaration of Helsinki’ (52nd WMA General Assembly, Edinburgh, Scotland, October 2000). Subjects were informed about the study orally and in writing, and signed a written informed consent before participation.

### Samples collection

Effluent samples were obtained from two healthy female ileostomy subjects (A and B), age 65 and 61, respectively. A total of 100ml effluent of each subject was stored in 100 ml RNAlater (Ambion, AM7021) using a 500 ml polypropylene copolymer centrifuge bottle (3120–0500; Nalgene Nunc International, USA) at room temperature for a minimum of 4 hours, before transported on dry ice. Afterwards, samples were aliquoted into portions of 20-30 ml in 50 ml tubes (T2068; Greiner Bio-One, The Netherlands) and stored at −80°C until further use.

### Total RNA and DNA extraction

Cell pellets were obtained from the RNAlater-effluent samples by adding 4 volumes of PBS, followed by centrifugation at 4600×g and 4°C for 10 minutes using Heraeus Multifuge 3 S-R Centrifuge (DJB Labcare Ltd., England, UK). The cell pellet was re-suspended in 500 μl ice-cold TE buffer (Tris–HCl pH 7.6, EDTA pH 8.0). Total RNA and DNA were extracted from the resuspended cell pellet according to the Macaloid-based RNA isolation protocol [43] with the use of Phase Lock Gel heavy (5 Prime GmbH, Hamburg) [44] during phase separation. The aqueous phase was subsequently split in two aliquots up to 300 μl, for RNA extraction and DNA isolation.

For the RNA extraction, the aqueous phase was purified using the RNAeasy mini kit (Qiagen, USA), including an on-column DNAseI (Roche, Germany) treatment as described previously [43]. Total RNA was eluted in 30 μl ice-cold TE buffer and the RNA quantity and quality were assessed using a NanoDrop ND-1000 spectrophotometer (Nanodrop Technologies, Wilmington, USA) and Experion RNA Stdsens analysis kit (Biorad Laboratories Inc., USA), respectively.

For total DNA extraction, the sample (300 μl aqueous phase) was pretreated with 3 μl RNAse A (10 mg/ml; Qiagen GmbH, Hilden, Germany) at 37°C for 15 minutes. Subsequent steps employed a modified version of the QIAamp DNA Stool Mini Kit (Qiagen GmbH, Hilden, Germany) protocol. Initially, 22.5 μl proteinase K (20 mg/ml; Ambion) and 300 μl buffer AL from QIAmp kit were added to the sample followed by incubation at 70°C for 10 minutes. After addition of 300 μl ethanol, the sample was transferred to a QIamp column and centrifuged (13,000×g, 1 minute, at room temperature), bound DNA on columns was washed subsequently with the AW1 and AW2 buffers from QIAmp kit, according to manufacturer’s instructions. Finally, the DNA was eluted with 30 μl Nuclease Free Water (Promega).

The relative overall density of the original microbial community in the effluent samples was estimated by 16S rDNA copy-numbers in the total DNA isolated, using quantitative PCR (qPCR) with Bact-1369F and Prok-1492R primers [45]. The qPCR was performed according to the previously described protocol [46], with initial denaturation at 95°C for 3 minutes, followed by 40 cycles of 95°C (15s), 56°C (30s), 72°C (30s), and a final extension at 72°C for 5 minutes. The qPCR reactions were carried out in Hard-Shell semi skirted clear 96 well plates (Bio Rad) sealed with Microseal B film (Bio Rad) in 25 μl volumes using IQ SYBR green supermix (Bio-Rad) according to the manufacturer’s instructions with 200 nM of forward and reverse primer and 12ng (sample A) or 5ng (sample B) of the total DNA as template. The qPCR was performed on a C1000TM Thermal Cycler (Bio-rad) with a CFX96 optic module (Bio-rad) employing CFX Manager 2.1 (Bio-rad) software for analysis.

### Microbial composition and activity profiling

For 16S rDNA based microbial composition profiling and 16S rRNA based microbial activity profiling, barcoded amplicons from the V1-V2 region of 16S rRNA genes were generated by PCR and reverse transcription PCR (RT-PCR). Both PCR and RT-PCR were performed using the 27F-DegS primer [27] that was appended with the titanium sequencing adaptor A and a 8 nt sample specific barcode [47] at the 5’ end, and a equimolar mix of two reverse primers (338R I and II [48]), that were 5’-extended with the titanium adaptor B.

PCRs were performed in a total volume of 100 μl containing 1× HF buffer (Finnzymes, Vantaa, Finland), 2 μl PCR Grade Nucleotide Mix (Roche, Diagnostics GmbH, Mannheim, Germany), 2 U of Phusion® Hot Start II High-Fidelity DNA polymerase, 500 nM of a forward and the reverse primer mix (Biolegio BV, Nijmegen, The Netherlands). PCRs were performed using a thermocycler GS0001 (Gene Technologies, Braintree, U.K.) using 0.2-0.4 ng/μl of template DNA. The amplification program consisted of an initial denaturation step at 98°C for 30s, 30 cycles of: denaturation at 98°C for 10s, annealing at 56°C for 20s and elongation at 72°C for 20s, and a final extension step at 72°C for 10min. RT-PCRs were performed using a one-step RT-PCR system (Access Quick, Promega, Leiden, The Netherlands) according to the manufacturer protocol, albeit with 30 amplification cycles instead of 40 and with amplification steps, consisting of denaturation at 94°C for 10s, annealing at 56°C for 20s and elongation at 68°C for 20s. The size of the PCR and RT-PCR products (~375 bp) was confirmed by gel electrophoresis, analyzing 5 μl of the reaction mixture on a 1% (w/v) agarose gel, containing 1× SYBR® Safe (Invitrogen, Carlsbad, CA, USA). PCR and RT-PCR products were purified with the High Pure Cleanup Micro Kit (Roche) using 10 μl Nuclease Free Water for elution, and amplicon yields were quantified using a NanoDrop ND-1000 spectrophotometer.

Purified (RT-) PCR products were mixed in equimolar amounts followed by running the amplicons on an agarose gel, band-excision, and purification by the DNA gel extraction kit (Millipore, Billerica, MA, USA). Purified amplicon pools (rDNA-PCR, and rRNA-RT-PCR) were pyrosequenced using a Genome Sequencer FLX in combination with titanium chemistry (GATC-Biotech, Konstanz, Germany).

The pyrosequencing data analysis was carried out with a workflow employing the Quantitative Insights Into Microbial Ecology (QIIME) pipeline [49] using settings as recommended in the QIIME 1.2 tutorial with the several modifications: reads were filtered for chimeric sequences using Chimera Slayer [50], and OTU clustering was performed with an identity threshold of 97%, using parameters as recommended in the QIIME newsletter of December 17^th^ 2010 (http://qiime.wordpress.com/2010/12/17/new-default-parameters-for-uclust-otu-pickers/). Additional data handling was done using in-house developed Python and Perl scripts. Taxonomic classifications was performed using the Ribosomal Database Project (RDP) classifier version 2.2 [51].

### Enrichment of mRNA, double stranded cDNA synthesis, and Illumina sequencing

The mRNA enrichment was performed by the removal of 16S and 23S rRNA using sequence-based capture probes attached to magnetic beads (MICROBExpressTM, Ambion, Applied Biosystem, Nieuwerkerk a/d Ijssel, The Netherlands) using the manufacturer’s protocols [7]. The enriched mRNA was quantified spectrophotometrically (NanoDrop) and its quality was assessed by microfluidics-based electrophoresis system (Experion RNA Stdsens).

Double stranded cDNA was synthesized using the Invitrogen’s SuperScript® Double-Stranded cDNA Synthesis kit (Invitrogen, 11917–010), with addition of SuperScript® III Reverse Transcriptase (Invitrogen, 18080–044) and random priming using random hexamers (Invitrogen, 48190–011) as described previously [9,11] followed by RNAse A (Roche, Germany) treatment, phenol-chloroform extraction, and ethanol precipitation. Double stranded cDNA was quantified using the NanoDrop 1000 spectrophotometer and verified by the sequencing provider (GATC Biotech, Konstanz, Germany) using an Agilent 2100 Bioanalyzer (Agilent technologies Inc., Waldbronn, Germany).

Single read (sample A) and paired-end mRNA-Seq Illumina Libraries (sample B) were constructed from double-stranded cDNA (ds cDNA) according to the ChiP protocol [52] with insert size between 200-300 bp. Sequencing was done with a Illumina Hiseq2000. Each sequencing library was barcoded and sequenced at 5pM concentration, using a single-end protocol for sample A (~38 million reads/sample) and a paired-end sequencing protocol for sample B (~75 million reads/sample).

A technical replicate of sample A (A-rep) was generated, including independent RNA extraction, mRNA enrichment, and ds cDNA synthesis, followed by single-end sequencing. The A-rep tagged sample library was barcoded and pooled with 3 others libraries before sequencing using a single-end flow cell lane of Illumina Hiseq2000, generating ~13 million single-end reads in total. The Illumina and the pyrosequencing reads were deposited with the Sequence Reads Archive (NBCI) under accession number SRP020487 and SRP023505, respectively.

### Bioinformatics analysis pipeline

Quality control of the raw sequencing reads was performed using the FastQC program (http://www.bioinformatics.babraham.ac.uk/projects/fastqc/). Quality scores (Q) were calculated based on the Illumina 1.9 encoding using the American Standard Code for Information Interchange (ASCII) values of 2 to 40 followed by removal of the reads that have a minimum average Phred score of 10.

To reduce the size for each dataset, reads with identical sequences were pooled into unique reads, coded based on their dataset source, and analyzed using the developed four steps bioinformatics pipeline (Figure 1).

#### Step 1: Removal of ribosomal RNA sequences

Removal of the rRNA sequences from the dataset was done using two iterative steps, first by applying the SortMeRNA software using the default rRNA database included in the software package, which consist of 16S, 23S, 18S, and 28S rRNAs [53]. The second step was performed on the remaining reads using BLASTN with a minimum alignment bit score of 54 using a filtering database consisting of complete ribosomal RNA loci, and tRNA sequences of bacteria, archaea and eukaryota taken from the NCBI and SILVA [54] databases. To remove residual sequencing reads derived from the phiX spike-in control sequences and the adaptor sequences employed in Illumina sequencing chemistry, the filtering database in addition contained phiX spike-in and adaptor sequences.

#### Step2: Taxonomic identity and functional assignment of mRNA reads

To determine the phylogenetic origin of sequence reads that passed the rRNA/tRNA filter (mRNA reads), filtered reads were aligned using BLASTN to a prokaryotic genome database consisting of 3,979 bacterial and archaeal full and draft genomes obtained from the ftp of National Center for Biotechnology Information (bacteria and bacteria_draft directories) (October, 2012) and 8 in house bacterial genomes (6 *Streptococcus*, 1 *Veillonella*, and 1 *Enterococcus* related species; van den Bogert, in preparation) isolated from ileostomy effluent [55]. According to a simulation experiment (Additional file 5: Figure S3A) minimum bit scores thresholds of 148, 110, and 74 can be used for phylogenetic and functional assignments at genus level (with >80% confidence level), phylogenetic and functional assignment at family level (with >80% confidence level), and for a reliable function (COG) assignment (with > 95% confidence level), respectively. To reduce the computational workload BLASTN searches were performed in two iterative steps. First read alignment results were obtained with MegaBLAST employing a default word size of 28 for an initial exact match, followed by reassignment of unaligned reads and reads with bit scores lower than 74 using the slower, more sensitive BLASTN algorithm with word size of 11 for an initial exact matches. The mRNA reads phylogenetic profiling at genus level was done with reads with a minimum bit alignment score of 148.

#### Step 3: Classification of mRNA in gene and intergenic reads

Genome assigned reads that had at least 50% of the alignment length located within the predicted open reading frame (ORF) of a gene were classified as gene assigned reads, while reads that do not match this criterion were classified as intergenic reads.

Significantly identified genes, which have read assignments above the threshold set, were determined by ranking the genes based on the number of read assignments, followed by selecting the top ranking genes that captured 95% of the assigned reads.

#### Step 4: Functional assignment

Predicted gene products of identified protein encoding genes were assigned to COGs [40] by blast searches against the COG database (NCBI, http://www.ncbi.nih.gov/pub/COG/COG) using an E-value <10^-6^ for COG assignments. The Kyoto Encyclopedia of Genes and Genomes (KEGG) function annotation [41] of the thus identified encoded proteins was performed using KEGG Automatic Annotation Server (KAAS; http://www.genome.jp/tools/kaas/) [56] using a default gene-set derived from 25 genomes with 15 additional bacterial genomes (Additional file 3: Table S6), based on a bi-directional best hit assignment method.

### Analysis of the unassigned mRNA reads

Unassigned mRNA reads were further analyzed by a BLASTX to the complete NCBI protein database, protein sequences of the MetaHIT database [35] and human small intestinal metagenome databases [4] using a minimum bit-score of 40 (Additional file 5: Figure S3B). Due to the size of the databases, BLASTX analyses were performed for 10% of these un-assigned reads for evaluation purposes. Detected proteins were annotated using the COG [40] and KEGG [41] databases as described above.

### Reproducibility and paired-end versus single-end sequence analyses

To investigate the reproducibility of mRNA-reads assignment to a gene, Pearson correlation coefficients were calculated for the different mRNA sets. The reproducibility of the procedure was assessed by estimating the correlation between the technical replicates of dataset A and the individual paired-end datasets (B-left and B-right). In addition, the percentage of paired-end reads that found or not found a match during the assignment was calculated to evaluate the potential added value of paired-end sequencing for reliable metatranscriptome analysis.

### Functional analysis and metabolic pathway mapping of the protein assigned reads

Relative gene expression levels were determined by counting the number of reads that were assigned to a particular protein-encoding gene. Normalization was obtained by dividing each gene count by the total mRNA read count of each dataset and multiplied by the average of the total mRNA read count across all datasets [57]. Functional COG distribution profiles were generated based on the reads abundance for total and genus specific COG assignment. Two representative reference genomes of each genus were selected to perform the COG functional categories profiles comparison. The selected representative genomes of intestine origin were in house *Streptococcus* and *Veillonella* genomes of isolates obtained from an ileostomy subject [55] and *Clostridium beijerinckii* NCIMB 8052; whereas the selected genomes of the non-intestinal representatives of the same genera were those of *S. pneumoniae* 670-6B, *V. parvulla* DSM 2008, and *C. botulinum* A2 str. Kyoto. Metabolic mapping of the metatranscriptome profiles was performed quantitatively by mapping the KEGG annotation of the identified protein sequences onto metabolic pathway maps using the iPath v2 module (http://pathways.embl.de/iPath2.cgi#). Gene expression levels of the metabolic pathways was indicated by the line width, which was determined from the log 2 values of the read count of KEGG annotated proteins. Reads with alignment bit-scores ≥74 were used to create the global metabolic activity maps and other functional interpretations.

### Computational details

Data processing was done by in-house python (version 2.6) scripts, except where the use of other software modules is indicated.

## Competing interests

The authors declare that they have no competing interests.

## Authors’ contributions

ML prepared and performed the experiments, participated in data analyses and wrote the manuscript. JRG developed the analysis pipeline and performed bioinformatics data analysis. MD contributed to validation analysis of the pipeline. BvdB and JB performed the microbial composition profiling. HS facilitated the research and gave input to the manuscript. ES directed the project. EGZ assisted in legal procedures for sampling approval, participated in samples collection, and supervised the research. PS designed and supervised the construction of the analysis pipeline and gave input to the manuscript. MK was involved in the experimental design, supervised the research, and gave input to the manuscript. All authors read and approved the final manuscript.

## Supplementary Material

Additional file 1: Figure S1 Quality measurement of total RNA for sample A and B. Total RNA quality was measured based on the 16S/23S ratio using Experion RNA Stdsens analysis kit. Click here for file

Additional file 2: Figure S2 Distribution of average Phred score of raw Illumina reads. The distribution was based on a minimum average Phred score of 30 (green), below 30 and a minimum of 20 (orange), below 20 and a minimum of 10 (yellow), and below 10 (red). Click here for file

Additional file 3 Supplementary Tables. Click here for file

Additional file 4 Supplementary methods. Click here for file

Additional file 5: Figure S3 Minimum bit score cut off for phylogenetic and functional assignment. The read assignments were performed using MegaBLAST (a) and BLASTX (b). Validation was performed using 10,000 random *in silico* reads of 100bp length generated from protein-encoding sequences of fully sequenced prokaryote genomes of NCBI. The reads were assigned to phylogenetic levels at species (Red diamond), genus (Green triangle), family (Blue circle), order (Orange asterisk), class (Pink circle), phylum (Skyblue plus), and to functional COG (Violet square). Click here for file

Additional file 6: Figure S4 Genus phylogenetic profiling of datasets A and B. Phylogenetic profiling of detected bacterial genera in the 16S rDNA and rRNA sequences obtained from pyrosequencing (a) and for mRNA reads obtained from Illumina sequencing (b). Only genera that contribute at least 2% to one of the profiles were represented. Click here for file

Additional file 7: Figure S5 Distribution of protein encoding gene coverage for each dataset. The genes were classified based on the percentage of coverage over the full-length gene. For each coverage percentage, the abundance of gene assigned mRNA reads was calculated and classified according to the bit score of ≥ 148 (blue), ≥ 110, but below 148 (light grey), and ≥ 74, but below 110 (dark grey). The gene abundance for each coverage percentage before and after removal of the spurious reads alignment was indicated in red and black lines. Click here for file

Additional file 8: Figure S6 Paired-end read validation of datasets B. Percentage of gene assigned read pairs based on the congruence of the taxonomic (a) and functional assignments (b) obtained from the separate analysis of the left and right reads of each read pair. Pair-end reads that only have one end assigned to a gene are depicted in black. Click here for file

Additional file 9: Figure S7 Comparison of metabolic pathways detected in datasets A and A-rep. Overlapping pathways that were detected in both datasets A and A-rep, are indicated in green lines and (additional) pathways only detected in the higher depth of analysis dataset A are indicated in red lines. The line width is indicative of the gene expression level and is based on the log-2 values of the number of reads assigned to individual functions. Metabolic pathways were generated using iPath v2 based on KEGG annotation. Click here for file
