# Supplementary figures and images for: A comprehensive metatranscriptome analysis pipeline and its validation using human small intestine microbiota datasets
Source: BMC Genomics. 2013 Aug 2;14:530. doi: 10.1186/1471-2164-14-530 (PMC3750648; doi:10.1186/1471-2164-14-530)

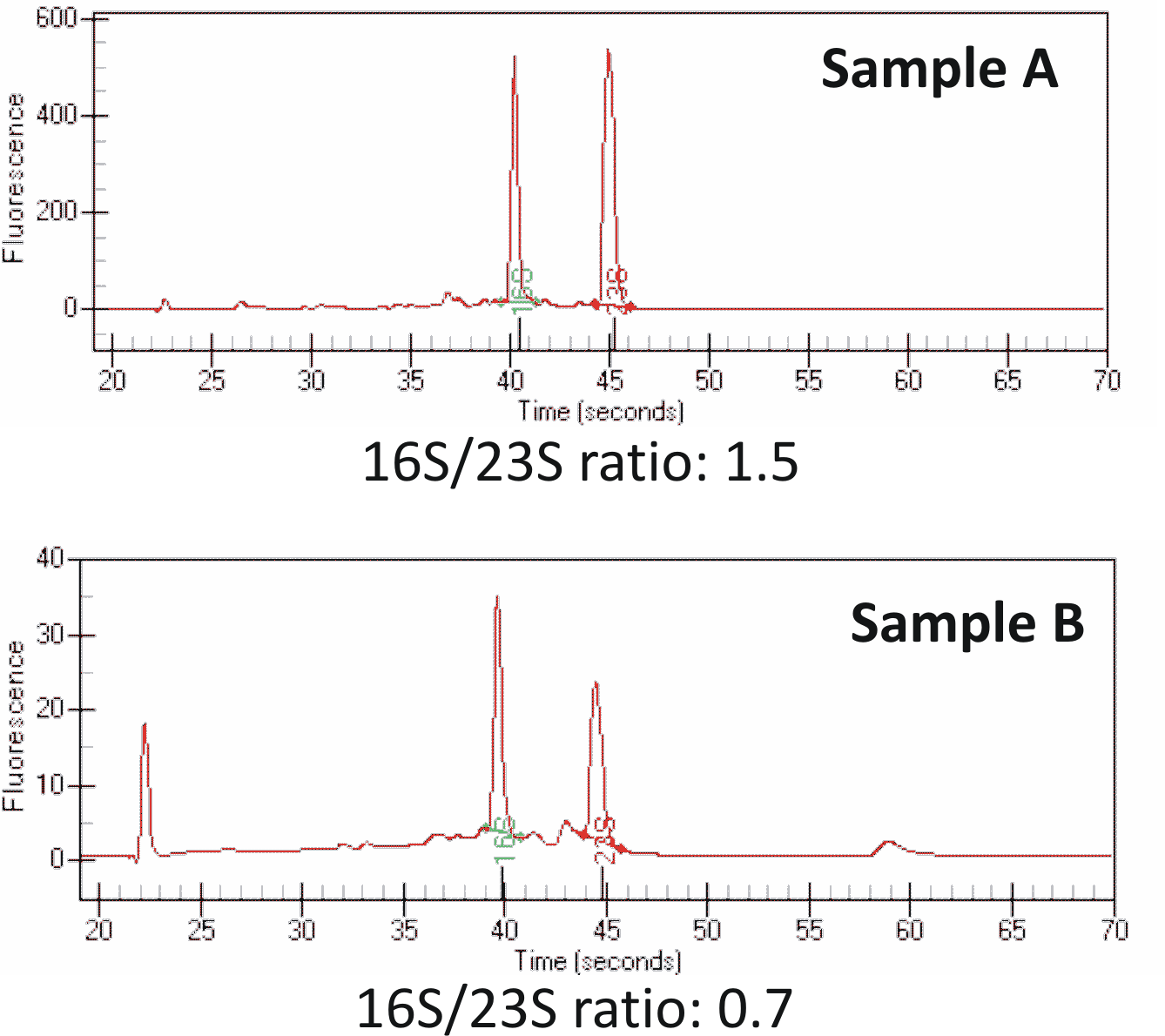

Supplement: Additional file 1: Figure S1 — Quality measurement of total RNA for sample A and B. Total RNA quality was measured based on the 16S/23S ratio using Experion RNA Stdsens analysis kit. [file 1471-2164-14-530-S1.png]

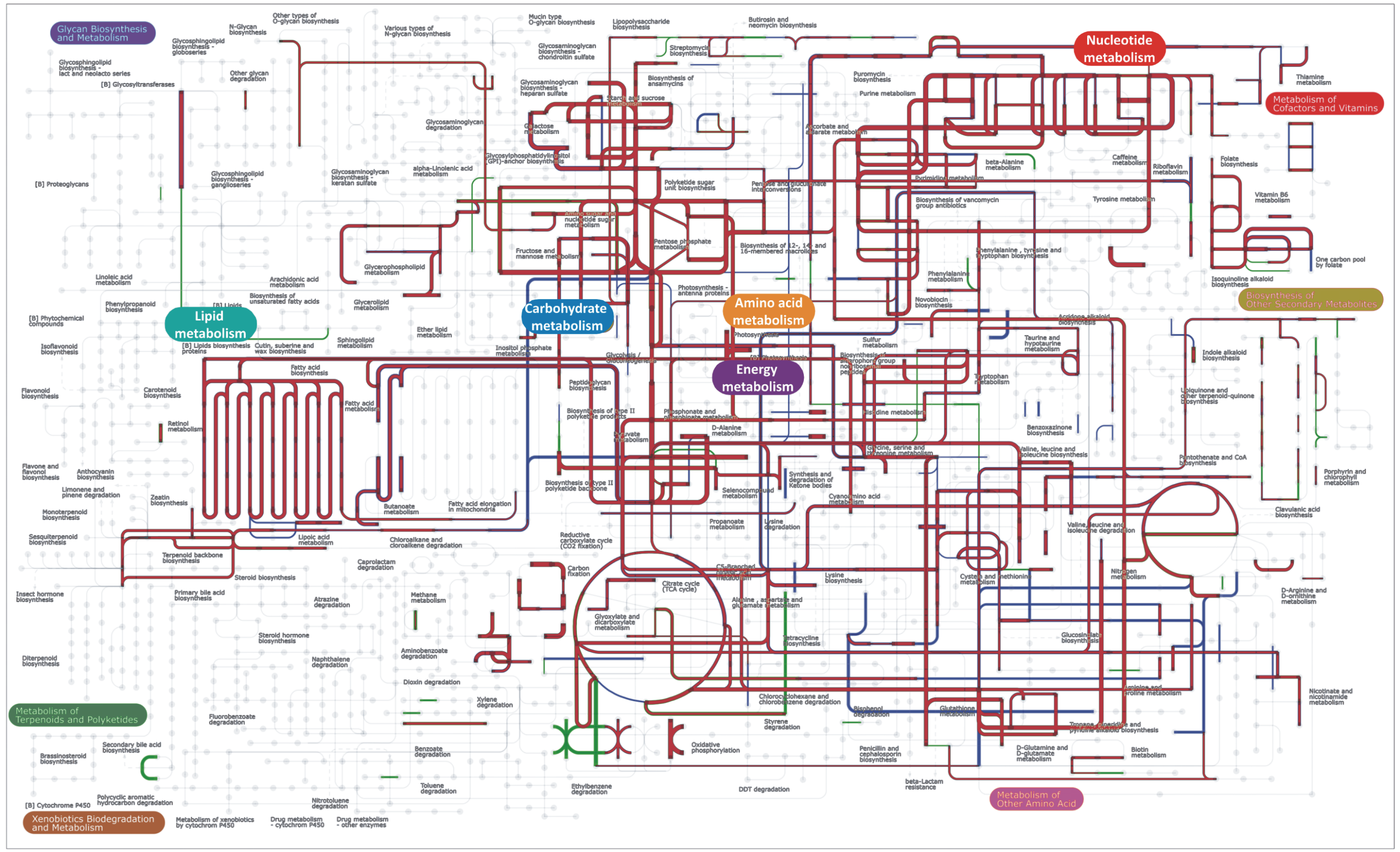

Supplement: Additional file 9: Figure S7 — Comparison of metabolic pathways detected in datasets A and A-rep. Overlapping pathways that were detected in both datasets A and A-rep, are indicated in green lines and (additional) pathways only detected in the higher depth of analysis dataset A are indicated in red lines. The line width is indicative of the gene expression level and is based on the log-2 values of the number of reads assigned to individual functions. Metabolic pathways were generated using iPath v2 based on KEGG annotation. [file 1471-2164-14-530-S9.png]
