# Supplementary material for: A comprehensive metatranscriptome analysis pipeline and its validation using human small intestine microbiota datasets
Source: BMC Genomics. 2013 Aug 2;14:530. doi: 10.1186/1471-2164-14-530 (PMC3750648; doi:10.1186/1471-2164-14-530)
Supplement: Additional file 3 — Supplementary Tables. [file 1471-2164-14-530-S3.docx]

**Table S1 - The number of Illumina sequencing reads after clustering.** Clustering was performed for identical Illumina reads in each dataset.

| **Dataset** | **Total reads (Q≥10)** | **Unique reads** | **% of compression** |
| --- | --- | --- | --- |
| A | 29,208,566 | 13,074,179 | 44,76 |
| A-rep | 8,534,765 | 3,442,837 | 40.34 |
| B-left | 42,006,183 | 12,520,920 | 29.81 |
| B-right | 41,410,328 | 12,307,590 | 29,72 |

**Table S2 – The number of reads after rRNA/tRNA removal.**

| **Dataset** | **Total reads (Q≥10)** | **rRNA/tRNA reads** | **mRNA reads** | **% of mRNA** |
| --- | --- | --- | --- | --- |
| A | 29,208,566 | 24,912,430 | 4,296,136 | 14.7 |
| A-rep | 8,534,765 | 6,838,506 | 1,696,259 | 19.9 |
| B-left | 42,006,183 | 36,075,240 | 5,930,943 | 14.1 |
| B-right | 41,410,328 | 35,494,380 | 5,915,948 | 14.3 |

**Table S3 – The number of alignments for each bit score from MegaBLAST and BLASTX validations.** The percentage of match was calculated based on the total alignments that have COG annotation in both input and reference genome. The NA column indicates alignments that have no COG annotation in the input, reference genome, or both.

| **MegaBLAST validation** | | | | | | | | | | | | | | | | | | | **BLASTX validation** | | | | | |
| --- | --- | --- | --- | --- | --- | --- | --- | --- | --- | --- | --- | --- | --- | --- | --- | --- | --- | --- | --- | --- | --- | --- | --- | --- |
| **Bit Score** | **Total alignments** | **Alignment at COG level** (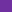) | | | | **Alignment at species level** (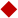) | | | | **Alignment at genus level** (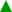) | | | | **Alignment at family level** (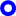) | | | | | **Bit Score** | **Alignment at COG level** (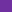) | | | | |
|  |  | Match | % Match | Mis-match | NA | Match | % Match | Mis-match | NA | Match | % Match | Mis-match | NA | Match | % Match | Mis-match | | NA |  | Match | % Match | | Mis-match | NA |
| 56 | 382323 | 141904 | 72.7 | 53176 | 187243 | 60856 | 15.9 | 321467 | 0 | 88340 | 23.4 | 289847 | 4136 | 118541 | 32.2 | 249589 | | 14193 | 32 | 1793760 | 90.3 | | 193410 | 725214 |
| 58 | 510420 | 219687 | 81.5 | 50010 | 240723 | 62520 | 12.2 | 447900 | 0 | 100840 | 20.0 | 403254 | 6326 | 152095 | 30.9 | 339480 | | 18845 | 33 | 2686897 | 91.5 | | 248458 | 1010483 |
| 60 | 422812 | 181626 | 84.1 | 34275 | 206911 | 68708 | 16.3 | 354104 | 0 | 102618 | 24.6 | 315196 | 4998 | 145631 | 35.6 | 262923 | | 14258 | 34 | 1809920 | 92.4 | | 149760 | 640942 |
| 62 | 399934 | 178655 | 86.8 | 27238 | 194041 | 63884 | 16.0 | 336050 | 0 | 98960 | 25.2 | 293917 | 7057 | 144993 | 37.7 | 239635 | | 15306 | 35 | 2698831 | 93.1 | | 200135 | 924719 |
| 64 | 418937 | 193057 | 88.8 | 24323 | 201557 | 61592 | 14.7 | 357345 | 0 | 99907 | 24.1 | 313994 | 5036 | 156529 | 38.6 | 248519 | | 13889 | 36 | 1798544 | 93.8 | | 119873 | 601487 |
| 66 | 378724 | 175213 | 90.1 | 19231 | 184280 | 60311 | 15.9 | 318413 | 0 | 101457 | 27.1 | 272801 | 4466 | 155663 | 42.5 | 210942 | | 12119 | 37 | 2675674 | 94.3 | | 162567 | 871258 |
| 68 | 369665 | 170458 | 91.8 | 15275 | 183932 | 60037 | 16.2 | 309628 | 0 | 101336 | 27.7 | 263978 | 4351 | 156760 | 43.7 | 201629 | | 11276 | 38 | 2633183 | 94.7 | | 146148 | 833476 |
| 70 | 366221 | 170204 | 92.7 | 13317 | 182700 | 60213 | 16.4 | 306008 | 0 | 103906 | 28.7 | 258178 | 4137 | 162670 | 45.7 | 192900 | | 10651 | 39 | 1735809 | 95.2 | | 88229 | 533818 |
| 72 | 345652 | 164196 | 94.1 | 10328 | 171128 | 56930 | 16.5 | 288722 | 0 | 101812 | 29.8 | 240077 | 3763 | 162443 | 48.3 | 173929 | | 9280 | 40 | 2540707 | 95.6 | | 117254 | 774487 |
| 74 | 367097 | 173396 | 94.8 | 9427 | 184274 | 60931 | 16.6 | 306166 | 0 | 112112 | 30.9 | 250870 | 4115 | 185519 | 51.9 | 171719 | | 9859 | 41 | 1658754 | 95.9 | | 71701 | 502743 |
| 76 | 348797 | 160587 | 95.3 | 7865 | 180345 | 67385 | 19.3 | 281412 | 0 | 118171 | 34.2 | 226915 | 3711 | 184029 | 54.2 | 155658 | | 9110 | 42 | 2426330 | 95.9 | | 102616 | 722896 |
| 78 | 334849 | 157485 | 96.0 | 6572 | 170792 | 56417 | 16.8 | 278432 | 0 | 108559 | 32.8 | 222745 | 3545 | 179547 | 54.9 | 147415 | | 7887 | 43 | 2368420 | 96.3 | | 89988 | 689967 |
| 80 | 325530 | 153428 | 96.6 | 5368 | 166734 | 54600 | 16.8 | 270930 | 0 | 108705 | 33.7 | 213467 | 3358 | 179977 | 56.6 | 137921 | | 7632 | 44 | 1535164 | 96.5 | | 55130 | 444132 |
| 82 | 360032 | 169203 | 97.1 | 4982 | 185847 | 59885 | 16.6 | 300147 | 0 | 126089 | 35.4 | 230583 | 3360 | 213902 | 60.7 | 138473 | | 7657 | 45 | 2229554 | 96.7 | | 75323 | 643294 |
| 84 | 328236 | 150206 | 97.1 | 4498 | 173532 | 59882 | 18.2 | 268354 | 0 | 118681 | 36.5 | 206596 | 2959 | 197748 | 61.5 | 123599 | | 6889 | 46 | 1437435 | 97.0 | | 43863 | 412223 |
| 86 | 321406 | 150120 | 97.6 | 3669 | 167617 | 58312 | 18.1 | 263094 | 0 | 118844 | 37.3 | 199966 | 2596 | 198610 | 63.1 | 116238 | | 6558 | 47 | 2106455 | 97.1 | | 63398 | 597793 |
| 88 | 321481 | 149948 | 97.9 | 3206 | 168327 | 56638 | 17.6 | 264843 | 0 | 120531 | 37.8 | 198136 | 2814 | 204039 | 64.8 | 110840 | | 6602 | 48 | 2020921 | 97.4 | | 54830 | 568417 |
| 90 | 380352 | 177818 | 98.2 | 3249 | 199285 | 67061 | 17.6 | 313291 | 0 | 149169 | 39.5 | 228212 | 2971 | 257174 | 68.9 | 116109 | | 7069 | 49 | 1308633 | 97.6 | | 31822 | 362559 |
| 92 | 328527 | 148238 | 98.1 | 2796 | 177493 | 67316 | 20.5 | 261211 | 0 | 135804 | 41.7 | 189772 | 2951 | 223523 | 69.3 | 99135 | | 5869 | 50 | 1900592 | 97.8 | | 43513 | 524737 |
| 94 | 318415 | 146612 | 98.1 | 2796 | 169007 | 61480 | 19.3 | 256935 | 0 | 130930 | 41.4 | 185174 | 2311 | 220317 | 70.4 | 92705 | | 5393 | 51 | 1214642 | 97.9 | | 25519 | 336005 |
| 96 | 319032 | 145709 | 98.1 | 2799 | 170524 | 62197 | 19.5 | 256835 | 0 | 135010 | 42.6 | 181551 | 2471 | 226981 | 72.4 | 86696 | | 5355 | 52 | 1767955 | 98.1 | | 33447 | 489542 |
| 98 | 419930 | 194489 | 98.6 | 2698 | 222743 | 78155 | 18.6 | 341775 | 0 | 186657 | 44.8 | 230039 | 3234 | 314857 | 76.2 | 98593 | | 6480 | 53 | 1687965 | 98.3 | | 28717 | 468090 |
| 100 | 325545 | 146235 | 98.5 | 2280 | 177030 | 66779 | 20.5 | 258766 | 0 | 146795 | 45.4 | 176420 | 2330 | 242222 | 75.5 | 78535 | | 4788 | 54 | 1081889 | 98.5 | | 16719 | 304829 |
| 102 | 319718 | 144198 | 98.7 | 1894 | 173626 | 68235 | 21.3 | 251483 | 0 | 148207 | 46.7 | 169179 | 2332 | 242794 | 77.1 | 72124 | | 4800 | 55 | 1550100 | 98.6 | | 22081 | 434995 |
| 104 | 325292 | 145397 | 98.5 | 2244 | 177651 | 71157 | 21.9 | 254135 | 0 | 154358 | 47.8 | 168311 | 2623 | 250274 | 78.1 | 70120 | | 4898 | 56 | 996014 | 98.7 | | 13478 | 280230 |
| 106 | 482025 | 219047 | 98.9 | 2408 | 260570 | 101868 | 21.1 | 380157 | 0 | 239276 | 50.0 | 239191 | 3558 | 390343 | 82.0 | 85629 | | 6053 | 57 | 1441399 | 98.8 | | 18245 | 406728 |
| 108 | 329701 | 145412 | 98.7 | 1953 | 182336 | 75285 | 22.8 | 254416 | 0 | 165011 | 50.4 | 162133 | 2557 | 264495 | 81.3 | 60707 | | 4499 | 58 | 1380824 |  | 98.8 | 16501 | 394819 |
| 110 | 328624 | 144801 | 98.8 | 1785 | 182038 | 77731 | 23.7 | 250893 | 0 | 169390 | 51.9 | 156711 | 2523 | 268032 | 82.6 | 56402 | | 4190 | 59 | 891048 |  | 98.9 | 9744 | 257405 |
| 112 | 334571 | 144938 | 98.9 | 1649 | 187984 | 85208 | 25.5 | 249363 | 0 | 179239 | 53.9 | 153012 | 2320 | 277022 | 83.7 | 53781 | | 3768 | 60 | 1301176 |  | 99.0 | 13679 | 378675 |
| 114 | 562488 | 251249 | 99.0 | 2561 | 308678 | 134824 | 24.0 | 427664 | 0 | 311404 | 55.8 | 246920 | 4164 | 485351 | 87.2 | 71378 | | 5759 | 61 | 840379 |  | 99.1 | 7679 | 247822 |
| 116 | 336587 | 146210 | 98.9 | 1635 | 188742 | 87885 | 26.1 | 248702 | 0 | 187949 | 56.2 | 146608 | 2030 | 286870 | 86.1 | 46179 | | 3538 | 62 | 1260468 |  | 99.2 | 10181 | 377458 |
| 118 | 341234 | 144759 | 98.9 | 1654 | 194821 | 95493 | 28.0 | 245741 | 0 | 197274 | 58.2 | 141621 | 2339 | 294921 | 87.3 | 42802 | | 3511 | 63 | 1301487 |  | 99.3 | 8927 | 402032 |
| 120 | 345779 | 146339 | 99.0 | 1540 | 197900 | 101239 | 29.3 | 244540 | 0 | 205269 | 59.7 | 138460 | 2050 | 302120 | 88.2 | 40227 | | 3432 | 64 | 893419 |  | 99.4 | 5128 | 281454 |
| 122 | 666583 | 294106 | 99.0 | 2886 | 369591 | 185889 | 27.9 | 480694 | 0 | 407270 | 61.4 | 255623 | 3690 | 604049 | 91.4 | 57134 | | 5400 | 65 | 1411237 |  | 99.4 | 7876 | 462823 |
| 124 | 352334 | 148590 | 98.9 | 1695 | 202049 | 110454 | 31.3 | 241880 | 0 | 221083 | 63.1 | 129380 | 1871 | 316239 | 90.5 | 33138 | | 2957 | 66 | 958355 |  | 99.5 | 4669 | 316879 |
| 126 | 342024 | 142334 | 98.9 | 1649 | 198041 | 105523 | 30.9 | 236501 | 0 | 213388 | 62.8 | 126630 | 2006 | 309060 | 91.1 | 30062 | | 2902 | 67 | 1414514 |  | 99.5 | 6729 | 490598 |
| 128 | 359222 | 147275 | 98.9 | 1621 | 210326 | 124428 | 34.6 | 234794 | 0 | 236403 | 66.1 | 121111 | 1708 | 328064 | 92.0 | 28552 | | 2606 | 68 | 1306581 |  | 99.6 | 5304 | 475081 |
| 130 | 814655 | 352889 | 99.1 | 3182 | 458584 | 269557 | 33.1 | 545098 | 0 | 543919 | 67.1 | 266533 | 4203 | 762862 | 94.3 | 46232 | | 5561 | 69 | 771566 |  | 99.7 | 2687 | 289376 |
| 132 | 357070 | 148363 | 98.9 | 1576 | 207131 | 126160 | 35.3 | 230910 | 0 | 240650 | 67.7 | 114995 | 1425 | 332021 | 93.6 | 22755 | | 2294 | 70 | 958871 |  | 99.7 | 3199 | 374598 |
| 134 | 349681 | 142816 | 98.9 | 1625 | 205240 | 125454 | 35.9 | 224227 | 0 | 238641 | 68.6 | 109337 | 1703 | 326477 | 94.0 | 21019 | | 2185 | 71 | 508139 |  | 99.6 | 1845 | 205359 |
| 136 | 382338 | 151875 | 99.0 | 1601 | 228862 | 157812 | 41.3 | 224526 | 0 | 274165 | 72.0 | 106656 | 1517 | 360796 | 94.9 | 19443 | | 2099 | 72 | 562678 |  | 99.7 | 1764 | 232327 |
| 138 | 994395 | 425845 | 99.2 | 3235 | 565315 | 385780 | 38.8 | 608615 | 0 | 714204 | 72.1 | 276654 | 3537 | 953793 | 96.3 | 36335 | | 4267 | 73 | 386850 |  | 99.6 | 1382 | 165131 |
| 140 | 368536 | 151253 | 99.1 | 1348 | 215935 | 153519 | 41.7 | 215017 | 0 | 268167 | 73.0 | 99004 | 1365 | 351733 | 96.0 | 14807 | | 1996 | 74 | 179133 |  | 99.6 | 640 | 78089 |
| 142 | 362679 | 147115 | 99.1 | 1379 | 214185 | 155196 | 42.8 | 207483 | 0 | 265899 | 73.6 | 95489 | 1291 | 347330 | 96.2 | 13602 | | 1747 | 75 | 179593 |  | 99.6 | 681 | 81496 |
| 144 | 412093 | 159461 | 99.0 | 1548 | 251084 | 204334 | 49.6 | 207759 | 0 | 319013 | 77.6 | 91830 | 1250 | 398045 | 97.0 | 12268 | | 1780 | 76 | 78653 |  | 99.7 | 255 | 37988 |
| 146 | 1254696 | 525523 | 99.3 | 3905 | 725268 | 580297 | 46.3 | 674399 | 0 | 960693 | 76.8 | 290700 | 3303 | 1224804 | 97.9 | 25846 | | 4046 | 77 | 71419 |  | 99.7 | 226 | 34886 |
| 148 | 386354 | 151824 | 99.1 | 1367 | 233163 | 194427 | 50.3 | 191927 | 0 | 302562 | 78.5 | 82694 | 1098 | 376041 | 97.8 | 8646 | | 1667 | 78 | 31217 |  | 99.7 | 103 | 15079 |
| 150 | 366962 | 145515 | 99.1 | 1252 | 220195 | 183743 | 50.1 | 183219 | 0 | 288118 | 78.8 | 77744 | 1100 | 357624 | 97.8 | 7919 | | 1419 | 79 | 25947 |  | 99.7 | 77 | 12986 |
| 152 | 489509 | 182706 | 99.1 | 1647 | 305156 | 294201 | 60.1 | 195308 | 0 | 407188 | 83.4 | 81275 | 1046 | 480914 | 98.6 | 7065 | | 1530 | 80 | 22333 |  | 99.5 | 115 | 12893 |
| 154 | 1612770 | 653793 | 99.3 | 4633 | 954344 | 883901 | 54.8 | 728869 | 0 | 1300390 | 80.8 | 308750 | 3630 | 1590187 | 98.9 | 18399 | | 4184 |  |  |  |  |  |  |
| 156 | 368503 | 143428 | 99.2 | 1224 | 223851 | 203925 | 55.3 | 164578 | 0 | 298958 | 81.4 | 68502 | 1043 | 362437 | 98.7 | 4750 | | 1316 |  |  |  |  |  |  |
| 158 | 360463 | 137409 | 99.0 | 1320 | 221734 | 203390 | 56.4 | 157073 | 0 | 294133 | 81.8 | 65427 | 903 | 355007 | 98.8 | 4373 | | 1083 |  |  |  |  |  |  |
| 160 | 748221 | 261548 | 99.4 | 1613 | 485060 | 553933 | 74.0 | 194288 | 0 | 672672 | 90.2 | 72935 | 2614 | 740211 | 99.4 | 4183 | | 3827 |  |  |  |  |  |  |
| 162 | 1981262 | 790630 | 99.3 | 5504 | 1185128 | 1192018 | 60.2 | 789244 | 0 | 1625597 | 82.2 | 352238 | 3427 | 1965076 | 99.4 | 12224 | | 3962 |  |  |  |  |  |  |
| 164 | 359544 | 136634 | 99.1 | 1199 | 221711 | 221216 | 61.5 | 138328 | 0 | 298070 | 83.1 | 60719 | 755 | 356111 | 99.3 | 2397 | | 1036 |  |  |  |  |  |  |
| 166 | 349252 | 130897 | 99.2 | 1115 | 217240 | 219887 | 63.0 | 129365 | 0 | 291174 | 83.5 | 57349 | 729 | 345985 | 99.3 | 2299 | | 968 |  |  |  |  |  |  |
| 168 | 337153 | 128389 | 99.1 | 1126 | 207638 | 212368 | 63.0 | 124785 | 0 | 282189 | 83.9 | 54294 | 670 | 334249 | 99.4 |  | 2018 | 886 |  |  |  |  |  |  |
| 170 | 3019253 | 1172771 | 99.3 | 7964 | 1838518 | 2017078 | 67.1 | 1002175 | 0 | 2517289 | 83.9 | 497694 | 4270 | 3003873 | 99.7 |  | 9803 | 5577 |  |  |  |  |  |  |
| 172 | 334767 | 123895 | 99.2 | 953 | 209919 | 228292 | 68.2 | 106475 | 0 | 281670 | 84.3 | 52538 | 559 | 332776 | 99.7 |  | 1111 | 880 |  |  |  |  |  |  |
| 174 | 324253 | 120621 | 99.2 | 1033 | 202599 | 223590 | 69.0 | 100663 | 0 | 275828 | 85.2 | 47873 | 552 | 322342 | 99.7 |  | 1022 | 889 |  |  |  |  |  |  |
| 176 | 3774891 | 1433258 | 99.4 | 8796 | 2332837 | 2727236 | 72.2 | 1047655 | 0 | 3189921 | 84.6 | 580449 | 4521 | 3762270 | 99.8 |  | 6802 | 5819 |  |  |  |  |  |  |
| 178 | 340802 | 122437 | 99.1 | 1064 | 217301 | 248581 | 72.9 | 92221 | 0 | 292877 | 86.0 | 47530 | 395 | 339297 | 99.8 |  | 650 | 855 |  |  |  |  |  |  |
| 180 | 320968 | 117361 | 99.3 | 846 | 202761 | 235924 | 73.5 | 85044 | 0 | 274775 | 85.7 | 45814 | 379 | 319769 | 99.8 |  | 563 | 636 |  |  |  |  |  |  |
| 182 | 324473 | 116859 | 99.2 | 993 | 206621 | 237431 | 73.2 | 87042 | 0 | 277718 | 85.7 | 46240 | 515 | 323188 | 99.8 |  | 597 | 688 |  |  |  |  |  |  |
| 184 | 5860127 | 2156234 | 99.4 | 12778 | 3691115 | 4459568 | 76.1 | 1400559 | 0 | 5027400 | 85.9 | 827206 | 5521 | 5844348 | 99.9 |  | 8101 | 7678 |  |  |  |  |  |  |
| 186 | 328825 | 116979 | 99.2 | 958 | 210888 | 250488 | 76.2 | 78337 | 0 | 284749 | 86.7 | 43592 | 484 | 327770 | 99.9 |  | 438 | 617 |  |  |  |  |  |  |
| 188 | 297853 | 108386 | 99.3 | 717 | 188750 | 225248 | 75.6 | 72605 | 0 | 255879 | 86.0 | 41720 | 254 | 297118 | 99.9 |  | 376 | 359 |  |  |  |  |  |  |
| 190 | 305023 | 107962 | 99.1 | 937 | 196124 | 230052 | 75.4 | 74971 | 0 | 263260 | 86.4 | 41559 | 204 | 304111 | 99.9 |  | 442 | 470 |  |  |  |  |  |  |
| 192 | 10810408 | 3955308 | 99.5 | 19914 | 6835186 | 8299204 | 76.8 | 2511204 | 0 | 9507718 | 88.0 | 1292424 | 10266 | 10786771 | 99.9 |  | 10526 | 13111 |  |  |  |  |  |  |
| 194 | 275331 | 101938 | 99.5 | 558 | 172835 | 211343 | 76.8 | 63988 | 0 | 241668 | 87.8 | 33471 | 192 | 274660 | 99.9 |  | 319 | 352 |  |  |  |  |  |  |
| 196 | 266777 | 98652 | 99.5 | 507 | 167618 | 203790 | 76.4 | 62987 | 0 | 232896 | 87.4 | 33621 | 260 | 266131 | 99.9 |  | 292 | 354 |  |  |  |  |  |  |
| 198 | 263462 | 94510 | 99.5 | 493 | 168459 | 200477 | 76.1 | 62985 | 0 | 232146 | 88.2 | 31090 | 226 | 262827 | 99.9 |  | 242 | 393 |  |  |  |  |  |  |
| 200 | 32622890 | 11951167 | 99.6 | 53451 | 20618272 | 26765625 | 82.0 | 5857265 | 0 | 30549942 | 93.8 | 2020448 | 52500 | 32484804 | 99.9 |  | 37368 | 100718 |  |  |  |  |  |  |

**Table S4 – mRNA reads assignment flow scheme.** Distribution of mRNA reads assignments to the reference genome database to protein-coding and non-coding sequences, within distinct bit-score ranges that were employed for phylogenetic assignment. Percentage was calculated on the number of mRNA reads of each assignment level.

| **Reads assignment to** | **mRNA reads classification** | **Number of reads for each dataset** | | | | | | | |
| --- | --- | --- | --- | --- | --- | --- | --- | --- | --- |
|  |  | **A** | **%** | **A-rep** | **%** | **B-left** | **%** | **B-right** | **%** |
| Genus (bit score ≥148) | Gene | 2,627,694 | 83.2 | 1,137,069 | 88.6 | 2,650,744 | 88.4 | 2,624,092 | 88.5 |
|  | Intergenic | 529,316 | 16.8 | 145,873 | 11.4 | 347,296 | 11.6 | 339,463 | 11.5 |
| Family (bit score 110-<148) | Gene | 402,148 | 84.6 | 183,413 | 90.7 | 761,115 | 86.4 | 762,670 | 86.3 |
|  | Intergenic | 73,097 | 15.4 | 18,856 | 9.3 | 119,630 | 13.6 | 121,145 | 13.7 |
| Function only (bit score 74-<110) | Gene | 208,702 | 85.9 | 79,535 | 89.2 | 655,363 | 87.6 | 662,237 | 87.6 |
|  | Intergenic | 34,331 | 14.1 | 9,630 | 10.8 | 93,192 | 12.4 | 93,355 | 12.4 |
| Unassigned reads | | 420,848 |  | 121,883 |  | 1,303,603 |  | 1,312,986 |  |

**Table S5 – Classification of protein encoding gene reads based on their functional annotation.** The protein encoding genes were classified based on their functional annotation obtained from COG databases. The COG category of “unknown function” was grouped as hypothetical genes. Percentage was calculated based on the total number of reads that were assigned to the protein encoding genes. For the BLASTX analysis only 10% of the unassigned reads from the core pipeline was used.

| **Protein encoding reads classification from the core pipeline** | | | | | | | | |
| --- | --- | --- | --- | --- | --- | --- | --- | --- |
| Number of : | A | | A-rep | | B-left | | B-right | |
|  | Number | % | Number | % | Number | % | Number | % |
| Reads assigned to genes with COG | 2,471,227 | 80.4 | 1,067,084 | 80.3 | 3,513,848 | 91.1 | 3,498,517 | 91.1 |
| Reads assigned to hypothetical genes | 603,184 | 19.6 | 262,133 | 19.7 | 343,114 | 8.9 | 342,728 | 8.9 |
| Unassigned reads from core pipeline | 420,848 |  | 121,883 |  | 1,303,603 |  | 1,312,986 |  |
| **Estimated unassigned reads after BLASTX** | | | | | | | | |
| Number of : | Number | % | Number | % | Number | % | Number | % |
| 10% random reads used for BLASTX | 40,490 |  | 12,669 |  | 130,583 |  | 130,750 |  |
| Reads assigned to genes with COG | 15,864 | 71.9 | 3,831 | 64.1 | 75,349 | 81.4 | 74,961 | 81.0 |
| Reads assigned to hypothetical genes | 6,191 | 28.1 | 2,144 | 35.9 | 17,194 | 18.6 | 17,582 | 19.0 |
| Reads assigned to MetaHIT and SI metagenome | 4,538 |  | 1,064 |  | 20,350 |  | 20,350 |  |
| The remaining unassigned reads | 13,897 |  | 6,694 |  | 38,040 |  | 38,207 |  |

**Table S6 - List of genomes used for KEGG annotation.** In addition to the default genomes, 15 bacteria genomes were added based on the representative for each genus available in KEGG database (grey).

| **Organism name** | **Code** |
| --- | --- |
| *Homo sapiens*  *Saccharomyces cerevisiae* (budding yeast)  *Escherichia coli* K-12 MG1655  *Salmonella enterica* subsp. *enterica* serovar Typhi CT18  *Haemophilus influenzae* Rd KW20 (serotype d)  *Pseudomonas aeruginosa* PAO1  *Neisseria meningitidis* MC58 (serogroup B)  *Helicobacter pylori* 26695  *Rickettsia prowazekii* Madrid E  *Mesorhizobium loti*  *Bacillus subtilis*  *Staphylococcus aureus* subsp. *aureus* N315 (MRSA/VSSA)  *Lactococcus lactis* subsp. *lactis* IL1403  *Streptococcus pneumoniae* TIGR4 (virulent serotype 4)  *Clostridium acetobutylicum* ATCC 824  *Mycoplasma genitalium*  *Mycobacterium tuberculosis* H37Rv  *Chlamydia trachomatis* D/UW-3/CX  *Borrelia burgdorferi* B31  *Synechocystis* sp. PCC 6803  *Aquifex aeolicus*  *Methanocaldococcus jannaschii*  *Archaeoglobus fulgidus*  *Pyrococcus horikoshii*  *Aeropyrum pernix* | hsa  sce  eco  sty  hin  pae  nme  hpy  rpr  mlo  bsu  sau  lla  spn  cac  mge  mtu  ctr  bbu  syn  aae  mja  afu  pho  ape |
| *Candida albicans*  *Klebsiella pneumoniae* subsp. *pneumoniae* MGH 78578  *Aggregatibacter aphrophilus*  *Streptococcus thermophilus* LMG18311  *Clostridium beijerinckii*  *Bifidobacterium dentium*  *Veillonella parvula*  *Rothia mucilaginosa*  *Campylobacter jejuni* subsp. *jejuni* NCTC 11168  *Listeria monocytogenes* EGD-e  *Lactobacillus plantarum* WCFS1  *Pediococcus pentosaceus*  *Enterococcus faecalis* V583  *Flavobacterium johnsoniae*  *Fusobacterium nucleatum* | cal  kpn  aap  stl  cbe  bde  vpr  rmu  cje  lmo  lpl  ppe  efa  fjo  fnu |
