# Supplementary material for: A comprehensive metatranscriptome analysis pipeline and its validation using human small intestine microbiota datasets
Source: BMC Genomics. 2013 Aug 2;14:530. doi: 10.1186/1471-2164-14-530 (PMC3750648; doi:10.1186/1471-2164-14-530)
Supplement: Additional file 4 — Supplementary methods. [file 1471-2164-14-530-S4.docx]

**Additional files for:**

**A comprehensive metatranscriptome analysis pipeline and its validation using human small intestine microbiota datasets**

**Milkha M Leimena^1,2^*, Javier Ramiro-Garcia^1,2,3^*, Mark Davids^1,3^, Bartholomeus van den Bogert^1,2^, Hauke Smidt^2^, Eddy J Smid^1,4^, Jos Boekhorst^6,7^, Erwin G Zoetendal^1,2^, Peter J Schaap^1,3§^, and Michiel Kleerebezem^1,2,5,7§^**

^1^TI Food and Nutrition (TIFN), P.O. Box 557, 6700 AN Wageningen, the Netherlands

^2^Laboratory of Microbiology, ^3^Laboratory of System and Synthetic Biology, Wageningen University, Dreijenplein 10, 6703 HB, Wageningen, the Netherlands

^4^Laboratory of Food Microbiology, Wageningen University, P.O. Box 8129, 6700 EV Wageningen, The Netherlands

^5^Host-Microbe Interactomics Group, Wageningen University, P.O. box 338, 6700 AH Wageningen, The Netherlands

^6^Centre for Molecular and Biomolecular Informatics, Radboud University Medical Centre, Nijmegen, Netherlands

^7^NIZO Food Research B.V., P.O. Box 20, 6710 BA, Ede, the Netherlands

**Determination of the bit score cut off for reads assignment to genomes**

For a read length of 100nt a maximal BLASTN alignment bit score of 198 can be obtained. To define the appropriate cut-off value for accurate phylogenetic and functional assignments, a set of *in silico* reads was generated, which consisted of 18,416,052 random fragments of 100bp length deriving from protein coding genes of 1754 completely sequenced prokaryote genomes obtained from NCBI database (June, 2012). These reads were given taxonomic and if available COG identifiers. The reads were aligned using MegaBLAST with default settings against the coding sequences of completely sequenced bacteria genomes with a max of 10 hits per query. In total, 85 million alignments (excluding self-hit) were generated, of which 8,664,954 (47%) have COG identifiers. For all hits the taxonomic ranks between the query and subject were compared and classified as a match or mismatch (Table S3). The same was done for the COG functional annotations with the exception that both the query and the subject needed a COG annotation. The results were binned based on the bit score of the alignment and the average percentage of matches was calculated (Figure S3A).

The analysis using MegaBLAST allowed precise assignments of the sequencing reads to a certain functional or taxonomic level depending on the alignment bit score. However, due to a high sequence similarity between species even at maximum bit score (198), an accurate assignment at species level still cannot be achieved. The highest phylogenetic classification with >80% confident level could be achieved at genus level using read alignment with a minimum bit score of 148, followed by assignment at family level using minimum bit score alignment of 110 (Figure S3A). Furthermore, all read alignments with minimum bit score of 74 could be reliably assigned to a COG-based function with >95% confidence level, which was important for biological interpretation of the metatranscriptome data.

In addition, an appropriate cut-off for COG functional assignment using BLASTX was validated using the same procedure as was performed for MegaBLAST, by taking the protein sequences of completely sequenced bacteria genomes obtained from NCBI database. BLASTX was performed using a total of 8,770,000 random *in silico* simulated reads of 1754 fully sequenced prokaryote genomes. In total of 88,929,281 alignments were generated, of which 68,167,048 could be matched for COG annotation. Using the BLASTX algorithm, bit-scores of 40 or higher allowed accurate COG assignments at a >95% confidence level (Figure S3B), and the bit-score >40 was selected as the cut-off for BLASTX assignment.

Additional validation was performed to support the cut-off value selection by performing MegaBLAST of 1 million *in silico* generated random reads to the complete and draft bacteria genomes of NCBI, resulting in only 4 read assignments to the genomes with bit score of 56 (below the cut off value). This indicated that it is not possible for a random read, which have no functional attributes, to gather an appropriate assignment within the bacteria genomes, thereby supporting the robustness of the 74-bit-score cut-off value for function assignment.
